# Scientometric Analysis and Mapping of Scientific Articles on Multiple Sclerosis-Related Neurogenic Lower Urinary Tract Dysfunction

**DOI:** 10.5152/tud.2023.23137

**Published:** 2023-11-01

**Authors:** Sakineh Hajebrahimi, Masoud Zeynalzadeh, Ashkan Shafigh, Helia Mostafaei, Moloud Balafar, Nima Naghdi, Hanieh Salehi-Pourmehr

**Affiliations:** 1 Research Center for Evidence-Based Medicine, Iranian EBM Centre: A JBICentre of Excellence, Tabriz University of Medical Sciences, Faculty of Medicine, Tabriz, Iran; 2 Student Research Committee, Tabriz University of Medical Sciences, Tabriz, Iran; 3 Emergency and Trauma Care Research Center, Tabriz University of Medical Sciences, Tabriz, Iran; 4 Department of Urology, Tabriz University of Medical Sciences, Faculty of Medicine, Tabriz, Iran

**Keywords:** Bibliometric analysis, multiple sclerosis, neurogenic urinary bladder disorder

## Abstract

**Objective:**

Patients suffering from multiple sclerosis (MS) frequently experience lower urinary tract (LUT) dysfunction, which significantly impacts their quality of life. This study’s objective was to conduct a scientometric analysis of the literature on MS-induced neurogenic LUT dysfunction.

**Methods:**

Using bibliometric methods, we examined the literature on neurogenic lower urinary tract dysfunction (NLUTD) in MS patients without restricting it to prevalence studies or specific management methods. We considered contributions from authors, organizations, nations/regions, as well as the evolution of theoretical frameworks, research subtopics, and influential papers. In January 2023, we searched the complete Scopus database, without imposing any language or date constraints, identifying relevant documents related to urology clinical investigations of MS-induced NLUTD. The original articles were categorized into 4 groups: narrative reviews, systematic reviews and meta-analyses, research of levels 1-4, and case reports/series.

**Results:**

On January 1, 2023, our search yielded 72 sources published between 1977 and 2022, including journals and books. The average time before publication was 11.2 years. Each document received an average of 18.1 citations, totaling 1.299 citations per year. The author's analysis explored relationships, productivity, and coauthorship networks among authors and institutions based on bibliographic records. Chartier-Kastler E, Karmonik C, and Khavari R ranked highest with 8 publications each. The University of Catania claimed the top position, followed by Houston Methodist Hospital and Paris University, recognized as the leading institutions in this field.

**Conclusion:**

An analysis of diagnosis, therapy, and rehabilitation of MS-related NLUTD may be helpful for future bibliometric research in the field to better direct output.

**Main Points**

- The study focuses on conducting a scientometric analysis of scientific articles related to Multiple Sclerosis (MS)-induced Neurogenic Lower Urinary Tract Dysfunction (NLUTD). Patients with MS often suffer from lower urinary tract dysfunction, impacting their quality of life.
- The study utilizes bibliometric methods to analyze a wide range of literature on NLUTD in MS patients. It examines contributions from authors, organizations, nations/regions, theoretical frameworks, research subtopics, and influential papers. A comprehensive search in the Scopus database was conducted in January 2023.
- The search yielded 72 relevant sources published between 1977 and 2022. On average, there was an 11.2-year gap before publication, and each document received an average of 18.1 citations. The study identifies influential authors, institutions, and journals in the field.
- MS-induced NLUTD significantly affects the quality of life, with symptoms often linked to the storage phase and urodynamic anomalies. Various factors, including spinal cord injuries and cognitive issues, contribute to these symptoms. Management strategies include peripheral tibial nerve stimulation, pharmaceutical intervention, and behavioral adjustment.
- The study identifies research hotspots, upcoming trends, and areas that require more attention, offering valuable insights for researchers, healthcare professionals, and students. It emphasizes the importance of conducting systematic analyses using bibliometric methods to understand and improve the field of MS-induced NLUTD.

## Introduction

The most prevalent progressive neurological condition in young people is multiple sclerosis (MS), with an occurrence rate of 108 cases for every 100 000 people in Europe and an average age onset of 30 years.^1,2^ Patients with MS frequently experience lower urinary tract (LUT) dysfunction, which significantly impacts their quality of life (QoL). The symptoms most commonly reported are associated with the storage phase, and the most prevalent urodynamic anomaly during this phase is detrusor overactivity.^3-7^ Over 80% of multiple sclerosis patients report experiencing symptoms related to lower urinary tract issues. Early neurological illness symptoms can appear and can occasionally be described at the time of initial presentation.^8^ According to clinical data, spinal cord injuries are the most common cause of lower urinary tract symptoms (LUTS), and there is an association between the severity of pyramidal symptoms affecting the lower limbs and these LUTS. However, cognitive issues such as memory loss, language impairment, and apraxia, as well as other localized factors such as bladder outlet obstruction, stress-related incontinence, or urinary tract infections, in addition to functional incontinence due to reduced mobility or extensive debilitation, or medication side effects, may also contribute to LUTS (such as opioid analgesics or tricyclic antidepressants). As a person’s handicap increases, both cognitive symptoms and LUTS tend to deteriorate over time, becoming increasingly challenging to address.^9^ Many researchers have written articles on MS-induced neurogenic LUT dysfunction (NLUTD). Despite the growing body of studies, there is currently no scientometric analysis on this issue.^10,11^ Scientometric analyses are more frequently utilized for funding allocation, carrier selection, and evaluation procedures.^12^ Moreover, scientometric analyses are useful for understanding the background and present state of a particular subject of research. Through the use of scientometric methods, we can obtain a dependable approach for mapping both the internal and external aspects of a scientific field by monitoring factors such as primary production, article content and quality, citation patterns, as well as the intentions of researchers as reflected in their published works. This study’s objective was to conduct a scientometric analysis of the literature on MS-induced NLUTD.

## Material and Methods

The literature on NLUTD in MS patients was examined using bibliometric methods, with ethical approval obtained from the Ethics Committee of Tabriz University of Medical Sciences (IR.TBZMED.REC.1400.706). We did not take into consideration a restriction to a prevalence study or any management method. Additionally, we overlooked the input provided by authors, nations/regions, organizations, and the evolution of theoretical frameworks, research subtopics, and influential publications within the field.

### Search Strategy

The thorough Scopus database search was conducted in January 2023, without any limitations on language or date. Search terms included the following: ((TITLE-ABS-KEY (“Multiple Sclerosis”) OR TITLE-ABS-KEY (“Disseminated Sclerosis”) OR TITLE-ABS-KEY (“MS (Multiple Sclerosis)”))) AND ((TITLE-ABS-KEY (“Neurogenic Bladder”) OR TITLE-ABS-KEY (“Neuropathic Bladder”) OR TITLE-ABS-KEY (“Bladder Neurogenesis”) OR TITLE-ABS-KEY (“Neurogenic Urinary Bladder”) OR TITLE-ABS-KEY (“Urinary Bladder Neurogenesis”) OR TITLE-ABS-KEY (“Urinary Bladder Neurogenic Dysfunction”) OR TITLE-ABS-KEY (“Neurogenic Dysfunction of the Urinary Bladder”). Scopus analysis restriction tools were employed to exclude publications in languages other than English, letters, editorials, retractions, meeting abstracts and proceedings, errata, and corrections from the dataset.

### Inclusion Requirements

The papers’ titles and abstracts that were discovered during the initial search were examined. Documents involving a urology-related clinical investigation of the MS-induced NLUTD were judged pertinent. The original articles were divided into 4 categories: narrative reviews, systematic reviews and meta-analyses, research levels 1-4, and case reports/series.

### Analysis

Scopus analysis techniques were employed to document the identified articles and references, along with details about the countries, authors, academic entities, and publications involved. We measured research output and influence by computing the number of research publications and the frequency of citations for each article, thus establishing rankings for key outcomes—such as author, country, institution, and journal. RStudio, with Biblioshiny tool (RStudio 2023.03.1+446, Boston), a Shiny application designed for Bibliometrix, as the analytical tool converted the author and institutional data, accounting for changes in author names and institutional designations, including subcategories. Author, institution, and journal production are taken into account while determining the top 10 lists. Countries are rated according to productivity, and we also aggregate data from various continents. The impact is used as a criterion to compile the top 10 articles in the respective field.

## Results

In all, 72 sources were found in our search on January 1, 2023, using the stated search method, including journals, books, etc., that were published between 1977 and 2022. On average, 11.2 years elapsed before publication. An average of 18.1 citations were made for each document, and 1.299 were made on average each year. A total of 87 original articles, 37 reviews, 2 brief surveys, and 2 notes were among the documents that were retrieved (Table 1).

### Analysis of Coauthors

**Figure 1.**
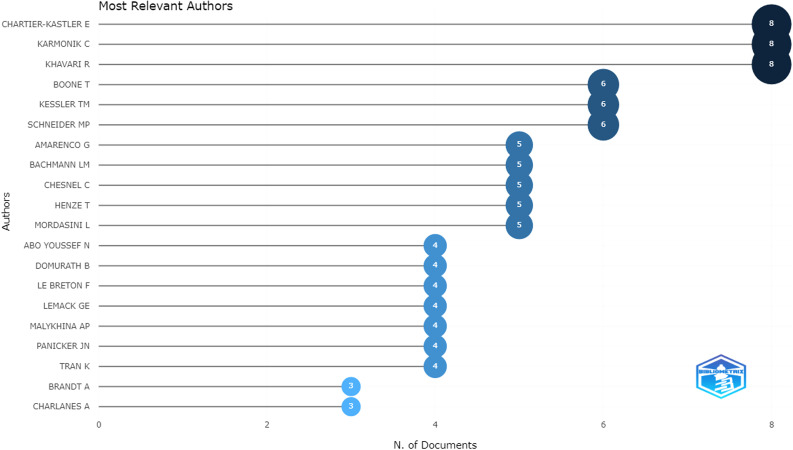
Most relevant authors.

Author analysis involves a thorough investigation of authors’ written works and their connections, encompassing authors’ productivity, their affiliations, and coauthor collaborations, as well as the networks formed by institutions, countries, or regions, as derived from bibliographic data. By statistically assessing a collection of journal papers, we identified the most prolific authors within the domain of MS-induced NLUTD. Figure 1 displays the writers in this field with the most publications.

According to the results, Chartier-Kastler E, Karmonik C, and Khavari R were in the first ranks with 8 publications. 

**Figure 2.**
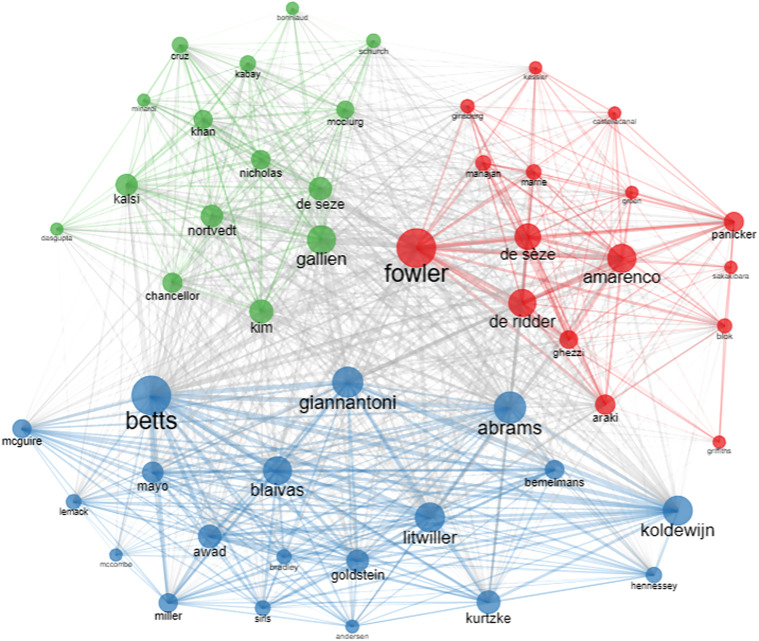
Author co-citation network.

The network of author co-citations is shown in Figure 2. Fowler CJ, Betts CD, Giannantoni A, Abrams P, Litwiller SE, and Koldewijn E.L. had the most co-citation networks, according to the results of the analysis.

Table 2 displays the authors who received the highest number of citations within the local context. The findings indicate that Henze T, De Ridder D, and Van Der AA F were the top authors in terms of local citations, accumulating 45, 33, and 33 citations, respectively.

**Figure 3.**
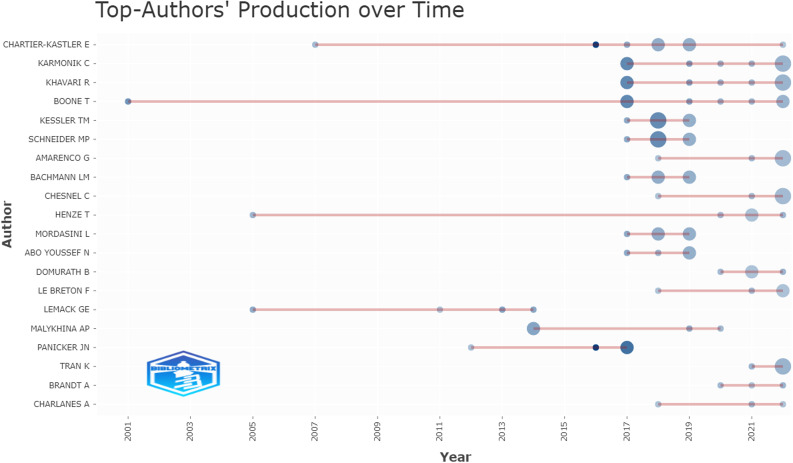
Top authors’ production over years.

The top author’s production over time is depicted in Figure 3. The earliest production belonged to Boone T (2001), and the recent active top authors in last year were Karmonik C, Khavari R, Chesnel C, and Tran K. 

### Country Collaboration

**Figure 4.**
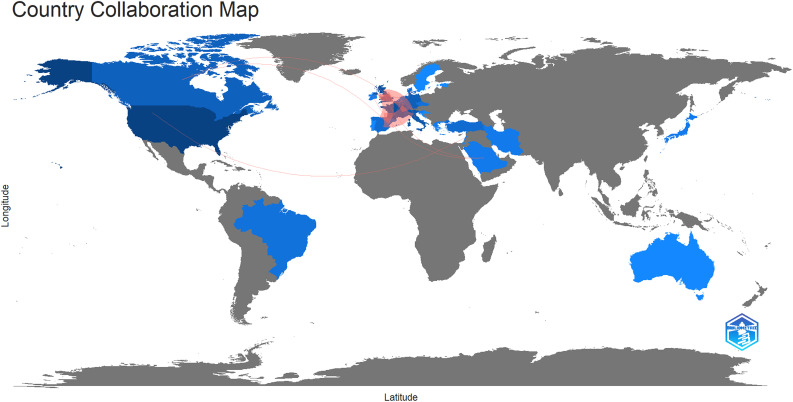
Country collaboration map.

In the realm of MS and research related to NLUTD, Europe emerged as the region with the most robust networks. Figure 4 illustrates the collaborative relationships among countries in this context.

**Figure 5.**
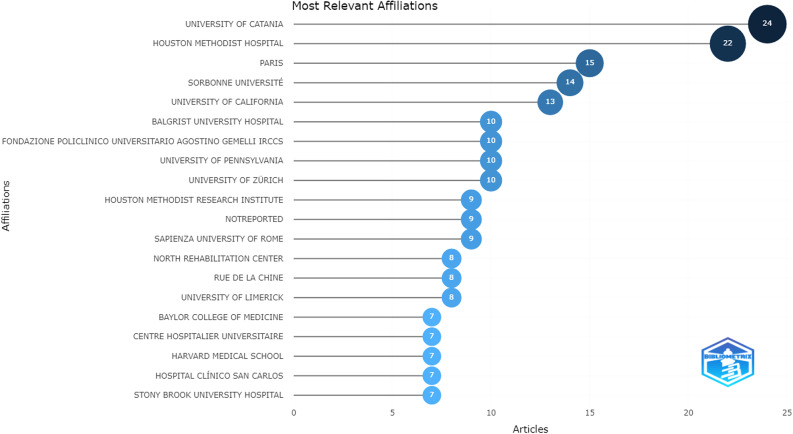
Most relevant affiliations.

Figure 5 displays the key affiliations that hold the utmost significance. The analysis indicates that the University of Catania occupied the leading position, while Houston Methodist Hospital emerged as another leading institution in this particular domain.

### Co-Word Evaluation

**Figure 6.**
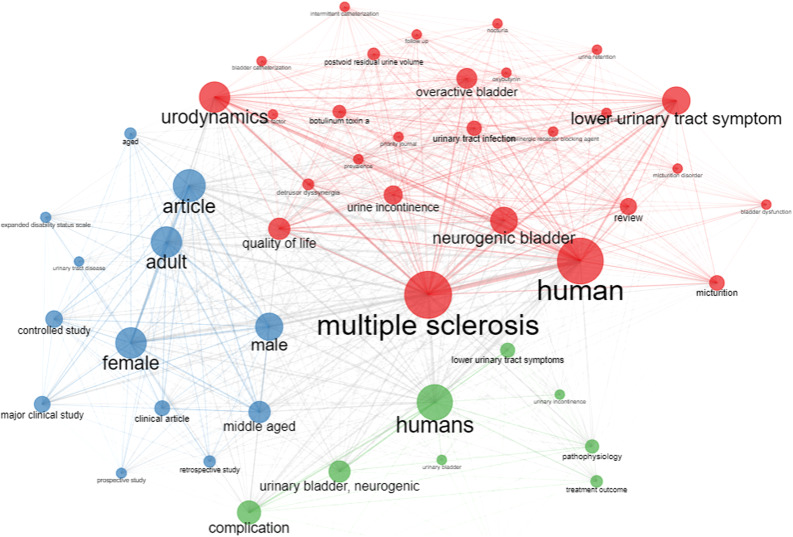
Co-occurring words network.

**Figure 7.**
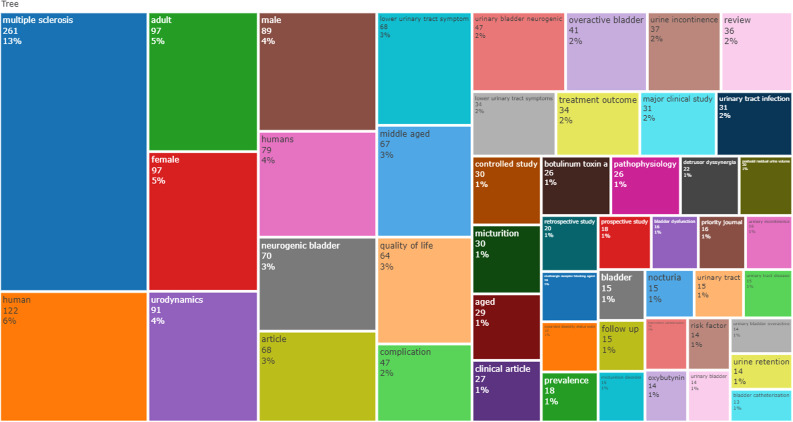
Keyword treemap.

We assessed the presence of specific keywords in the searched subjects through co-word analysis, which also revealed the connections between the searched keywords. Figure 6 illustrates the network of co-occurring words, while Figure 7 showcases a keyword treemap. Among the 50 nodes, the words “multiple sclerosis,” “human,” “adult,” “female,” “urodynamic,” and “lower urinary tract symptoms” were the most commonly encountered.

### Source Analysis

**Figure 8.**
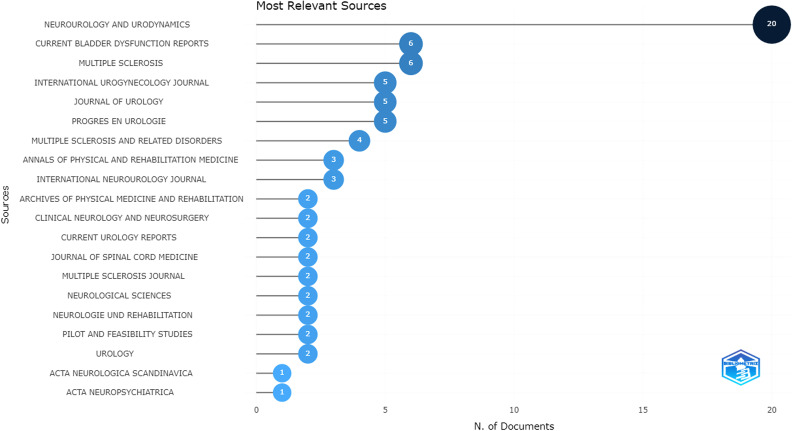
Most relevant sources.

*Neurourology and Urodynamic Journal*, followed by *Current Bladder Dysfunction Reports*, and *Multiple Sclerosis Journal* were the primary sources of significance in the context of MS-induced NLUTD, with 20, 6, and 6 published articles, respectively (Figure 8).

**Figure 9.**
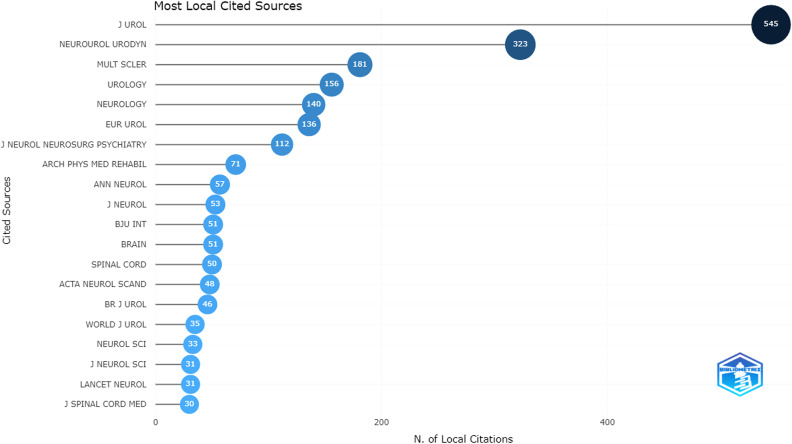
Most local cited sources.

Most local cited sources are depicted in Figure 9. According to the depicted sources, *The Journal of Urology* with 545 citations and the *Neurourology and Urodynamic*with 323 citations achieved the first and second ranks, respectively. 

**Figure 10.**
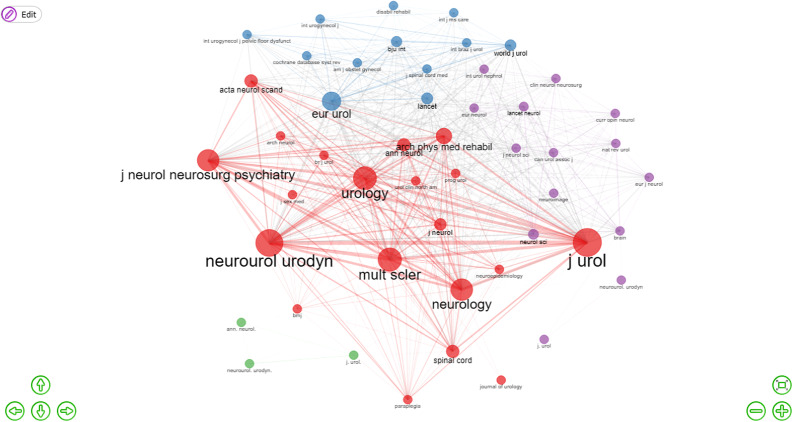
Sources of co-citation networks.

The sources of co-citation network are presented in Figure 10. According to the results, *The Journal of Urology*, *Neurourology and Urodynamics*, *Multiple Sclerosis Journal*, *Urology*, *Neurology*, and *Journal of Neurology*, *Neurosurgery and Psychiatry*, and *European Urology *had the strongest co-citation networks. 

**Figure 11.**
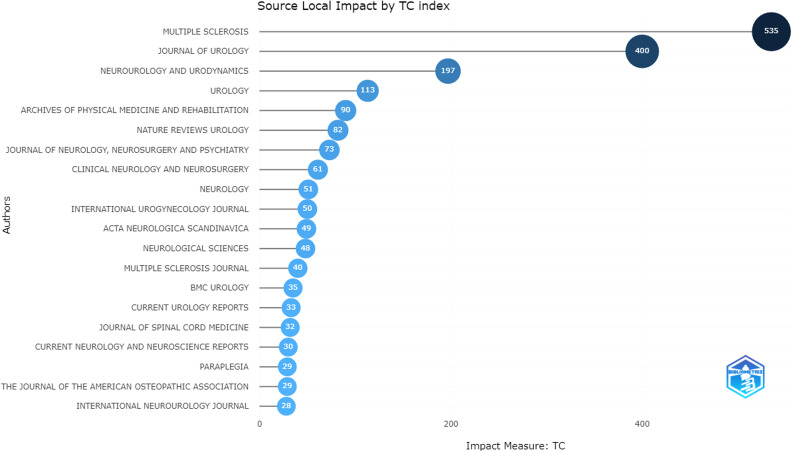
Sources’ impact by total citations.

The sources’ impact by total citations is illustrated in Figure 11. The *Multiple Sclerosis Journal* was the first journal to have highest impact by total citation.

### Subject Evaluation

Table 3 represents the document with the highest worldwide citation count. The article with the highest number of citations, authored by de Sèze and published in the *Multiple Sclerosis Journal *in 2007, accumulated a total of 294 references.

**Figure 12.**
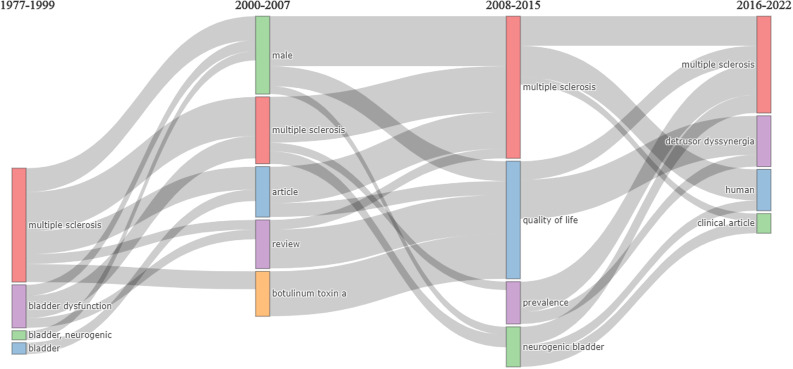
Thematic evolution.

**Figure 13.**
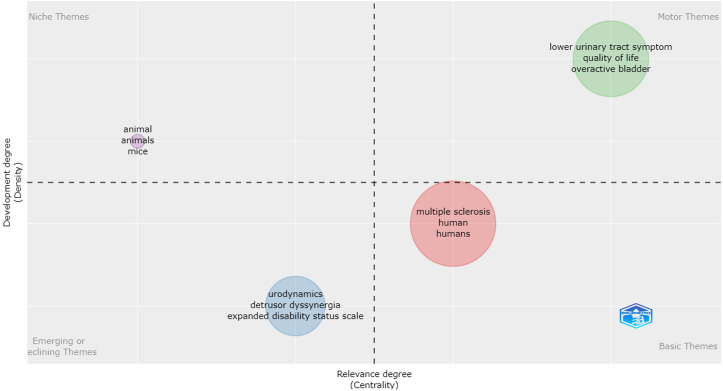
Thematic map.

In Figures 12 and 13, the thematic evolution and plan based on the year category are represented. In recent years, the most common themes were “multiple sclerosis,” “detrusor dyssynergia,” “human,” and “clinical articles.” Furthermore, the keywords “multiple sclerosis,” “human,” and “humans” were identified as basic themes that have not been adequately addressed.

**Figure 14.**
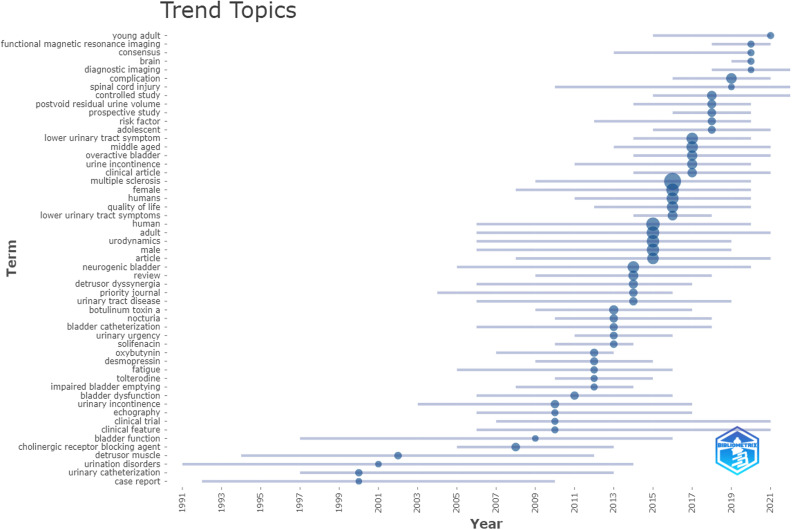
Trend topics.

The trend topics in the field of NLUTD in MS patients are illustrated in Figure 14. According to the results, in recent years, “young adult” and “functional magnetic resonance imaging,” along with “consensus” and “brain” and “diagnostic imaging,” occupied the first ranks. 

## Discussion

The demand for research teams, health-care professionals, and students to select the most pertinent literature and stakeholders grows as the amount of research on neurourological illnesses rises. Traditional reviews may not offer the same visual appeal and comprehensiveness as systematic assessments utilizing bibliometric methods, which encompass all publications and materials related to this subject. This method offers the opportunity to present visual information, allowing researchers who lack prior familiarity with the subject to gain a deeper understanding of the fundamental patterns within the area of investigation. It can also draw attention to recent research hotspots, upcoming trends, and ground-breaking studies. Over the course of the investigation, it was found that a wide variety of research articles on the MS and LUTD have been published, and the number of publications is growing every day. It might be helpful and useful to analyze these publications in this context to reveal trends and characteristics about authors, countries, topics, journals, and endeavors within the realm of medicine, particularly in the pursuit of disseminating scientific evidence.

### Impact of Multiple Sclerosis on Lower Urinary Tract

Up to 80% of MS patients experience NLUTD at some point during the course of their illness.^13^ The QoL of MS patients may be impacted by NLUTD, potentially leading to difficulties in both retaining urine (incontinence) and voiding the bladder (retention), which have a severe impact on their general well-being. Neurogenic detrusor overactivity (NDO) refers to a condition marked by uncontrolled contractions of the detrusor muscle, leading to symptoms like frequency, urgency, urge incontinence, and minimal or absent leftover urine after voiding, that is typically associated with lesions above the pontine micturition center. Frequency refers to the requirement to urinate often, whether it occurs during the daytime or at night (also known as nocturia), or both situations. Urgency denotes an abrupt and intense feeling of needing to urinate. Urge incontinence pertains to the unintentional leakage of urine that occurs when one is experiencing the sensation of urgency. Detrusor and external sphincter dyssynergia associated with NDO commonly manifest as urgency and/or urge incontinence with varying amounts of residual urine after voiding, which is frequently accompanied by pelvic floor stiffness and is indicative of medullary lesions located between the pons and the sacral micturition center. An absence or reduction in detrusor activity (urinary retention) brought on by incompetent urethra and/or weak pelvic floor muscles may result from infrasacral injuries affecting the conus.^14^ Up to 60% of MS patients experience NDO (urgency and urge incontinence);^15^ dysfunction of the detrusor sphincter coordination is observed in 35% of cases,^16^ while reduced detrusor muscle activity was observed in 25%.^17^ Bladder diseases that are left untreated can result in renal impairment, psychological anguish, sleep issues, social seclusion, and a deterioration in the quality of life.^13,18^ A multimodal and interdisciplinary strategy is necessary for the management of NLUTD, which may also help the improvement of the symptoms.^19^

### Management of Neurogenic Lower Urinary Tract Dysfunction

Peripheral tibial nerve stimulation, electrical therapies, pharmaceutical intervention, behavioral adjustment, pelvic floor muscle training, and intermittent catheterization are some of the more common forms of conservative treatment, or rehabilitation, that are used in this field. They also include lifestyle interventions, complementary therapies, anti-incontinence devices, and pads.^17,20^ The aim of rehabilitation therapy is to lessen urine incontinence or urgency while facilitating the emptying of the bladder. Achieving this goal necessitates the crucial task of striking an appropriate equilibrium in the management of bladder retention and NDO symptoms.

### Scientometry of Neurogenic Lower Urinary Tract Dysfunction Studies

Another challenge that the increasing amount of research on neuro-urinary diseases poses to health care professionals, students, and research teams is the selection of the most appropriate texts. Standard reviews may not be as visually appealing as systematic analyses of all publications and related materials on the subject using bibliometric analysis. This approach can provide visual data that helps researchers who are unfamiliar with the subject gain improved insight into the interconnected patterns within the studied field.

Based on the results of the current study, the most affiliations in the subject of MS and NLUTD are related to the United States, followed by Canada and European countries. Also, countries like Turkey, Iran, Saudi Arabia, and Australia have significant affiliations in the mentioned field. In the conducted studies, keywords such as multiple sclerosis, neurogenic complication, urodynamics, lower urinary tract symptom, and bladder have been searched together. Also, by looking at the treemap of the searched words, it can be seen that, in addition to the aforementioned keywords, other items such as treatment outcomes, QoL, follow-up, prevalence, bladder catheterization, oxybutynin, and botulinum toxin have been searched in this field’s research. A look at the graph of the evolution of searched keywords also confirms that although keywords like multiple sclerosis have always been of interest, researchers are more interested in other fields like detrusor dyssynergia, neurogenic bladder, and QoL in recent years. 

The graph related to the trend topics in the field of NLUTD related to MS also shows that in recent years, the topics of “brain” along with “consensus” and “functional magnetic resonance imaging” have been ranked first. The findings indicate that in recent years, most of the attention in this field has been focused on investigating the lower urinary tract complications caused by MS, as well as providing appropriate diagnostic methods and new treatments in order to increase the QoL of patients.

Two of the most frequently cited publications in our search were reviews.^17,21^ One of them centered on determining the factors that increase the likelihood of urinary tract issues in individuals with MS. The aim was to provide knowledgeable insights that could contribute to the development of useful guidelines for monitoring neurogenic bladder issues in MS, with the ultimate goal of enhancing prevention strategies and patient care. The 4 primary risk factors associated with upper urinary tract injury are the sustained elevation of detrusor pressure, the utilization of an indwelling catheter, the length of time 1 has multiple sclerosis, and intense neurogenic detrusor contractions with high amplitudes. Age above 50 years, male sex, and detrusor–sphincter dyssynergia may create 3 extra risk factors. In line with the methods advised by the French Health Authorities, suggestions for conducting urological follow-up over an extended period that take these particular hazards into consideration are constructed.^17^ The second most-cited document provides an updated reference that describes the cutting-edge method for treating multiple sclerosis from a neurological and urological perspective, with a focus on the pathophysiology, impact on the genitourinary system, diagnostic evaluation, and approaches to treating neurological and urological symptoms. A meta-analysis was conducted on 22 trials that included 1882 patients from clearly defined MS populations to assess the urodynamic results. The findings indicated that genitourinary symptoms, such as urgency, urge incontinence, frequency, and urine retention, are common in MS patients. The symptoms parallel pyramidal tract dysfunction but do not fully represent the underlying urological illness. A key factor in choosing the best bladder management strategy is urodynamic assessment. Detrusor hyperreflexia is the most frequent urodynamic finding in these individuals (62%). Subsequently, detrusor–sphincter dyssynergia is observed in 25% of patients, while hypocontractility is detected in 20% of cases. Renal impairment affects less than 1% of patients, and the majority can be managed with conservative treatments. New techniques for bladder restoration and diversion may be utilized successfully if more conservative procedures fail. Up to 80% of males and 72% of females experience sexual dysfunction, with therapeutic efforts directed toward addressing issues related to erectile or orgasmic dysfunction and overall disability improvement.^21^ Since MS-related NLUTD research is still largely centered in the several sectors in which it historically emerged, few publications are appearing in novel domains, indicating the commencement of interest. As researchers from many different fields delve into a topic during its growth phase, they uncover fresh applications facilitated by advancements in technology. A lot of funding is attracted by the expanding understanding of how the MS affects LUT function, which promotes the release of new research geared toward management technique.

Keywords are used to identify hotspots and trends in a subject field. Article keywords can offer crucial information regarding the topic or main argument of a specific study.^22^ Using keyword co-occurrence analysis provided a typical overview of the high-frequency terms multiple sclerosis, urodynamic, and neurogenic lower urinary tract dysfunction. By aiding knowledge acquisition and advancement, scientometric evaluations of fields aim to promote extra research and collaboration. When conceptualizing, research teams should consider the top publications and contributions from stakeholders while also bearing in mind the crucial topics within the field. Our study did not prioritize innovations or exploratory investigations, as all citations in this domain were identified through the selected keywords. Consequently, 1 limitation of our current study lies in the possibility that original contributions from institutions dedicated to discovery or innovation may not have been captured. The promising point is that, with the growing understanding of the effects of MS on LUT performance, many financial resources are attracted to research projects in this field. Regarding the strengths and advantages of conducting the present study, it should be said that a drawback of the existing research lies in the potential oversight of significant contributions made by institutions dedicated to exploration and pioneering endeavors. Burst keywords unveil significant changes in research over the past decade, shedding light on important directions in studies during this period. Moreover, they are good research topics for teams conducting fieldwork.^23^

The most recent domains to emerge, such as multiple sclerosis, NLUTD, urodynamics, and clinical trials, maybe the best choices because it may get harder to innovate in each one over time. 

### Limitations

The limitations of this study included its use of a single Scopus search and its categorization restrictions, although earlier research has demonstrated that studies confined to the Scopus database tend to compile records that accurately represent various domains due to the database’s extensive coverage of influential journals with high impact factors. Scopus contains advanced features to monitor, assess, and visualize research. It provides an in-depth examination of global research production across the fields of medicine, technology, science, arts, social sciences, and humanities.^24^ However, it is possible that some pertinent publications were overlooked because the study was restricted to journals that were indexed in the database. Restrictions on language and article types were put in place: non-English articles, editorials, comments, and conference proceedings—all of which have an impact on citation rates—were all disallowed. There was a minimal probability that their exclusion would have significantly changed the outcome because these literary genres are not well-liked or commonly referenced due to the built-in prejudice in the literature. The citation metrics were one of the handful of those that were used in this research. Since this research employed a limited set of citation metrics, it is important to note that rankings could differ when employing alternative metrics commonly found in the literature. 

### Strengthens of this Study and Recommendation for Future Research

The findings from the reviewed studies provide fundamental information accessible to researchers, university administrators, and research and technology policymakers. With an awareness of the state of articles in the medical field, specifically concerning multiple sclerosis and its impact on LUT, they can strategize increased investment in these areas and foster national and international collaborations. Given the significance of scientific articles in identifying critical medical topics, efforts should be focused on enhancing the quality of these articles, expanding global partnerships, and selecting appropriate journals for better dissemination of their results. Additionally, beyond expanding knowledge for decision-making, writing articles also contributes to the stability and widespread dissemination of research findings. The journals *Neurourology* *and Urodynamics*, *Current Bladder Dysfunction Report*, and *Multiple Sclerosis Journal* all published articles about NLUTS after MS research. The majority of the research listed were reviewed, and based on our findings using a search approach and a solitary database query, it seems that there is still potential for incorporating additional research methodologies like diagnostic tests and randomized controlled trials. The direction of future research should be decided upon in light of the significance of the topic. 

It is important to keep up with advancements in fields in order to develop effective research strategies and anticipate the needs of scientists. By analyzing the representations, maps, and models used in this study, which were derived from an examination of respected scientific publications worldwide, we can gain insights into areas where research is lacking and identify future requirements. Analysis of the diagnosis, therapy, and rehabilitation of MS-related NLUTD may be helpful for future bibliometric research in the field to better direct output.

## Figures and Tables

**Table 1.** Main information about data

**Description**	**Results**
**Main information about data**	
Time span	1977-2022
Sources (journals, books, etc.)	72
Documents	129
Average years from publication	11.2
Average citations per document	18.1
Average citations per year per document	1.299
References	4019
**Document types**	
Article	87
Conference paper	1
Note	2
Review	37
Short survey	2
**Document contents**	
KeyWords Plus (ID)	1139
Author’s keywords (DE)	247
**Authors**	
Authors	550
Author appearances	722
Authors of single-authored documents	8
Authors of multiauthored documents	542
**Authors collaboration**	
Single-authored documents	8
Documents per author	0.235
Authors per document	4.26
Coauthors per document	5.6
Collaboration index	4.48

**Table 2.** The most locally cited authors

**Author**	**Local Citations**
Henze T	45
De Ridder D	33
Van Der AA F	33
D'Hooghe M-B	31
Debruyne J	31
Dubois B	31
Guillaume D	31
Heerings M	31
Ilsbroukx S	31
Medaer R	31
Nagels G	31
Seeldrayers P	31
Van Landegem W	31
Willekens B	31
Zicot A-F	31
Chartier-Kastler E	26
Bachmann LM	22
Kessler TM	22
Mordasini L	22
Schneider MP	22

**Table 3.** The most global cited document

**Paper**	**DOI**	**Total Citations**	**Total Citations per Year**	**Normalized Total Citations**
DE SÈZE M, 2007, MULT SCLER	10.1177/1352458506075651	294	17.2941	1.947
LITWILLER SE, 1999, J UROL	10.1016/S0022-5347(01)61760-9	223	8.92	2.3069
BRADY CM, 2004, MULT SCLER	10.1191/1352458504ms1063oa	193	9.65	2.9242
GOLDSTEIN I, 1982, J UROL	10.1016/S0022-5347(17)53037-2	148	3.5238	1.8974
PHÉ V, 2016, NAT REV UROL	10.1038/nrurol.2016.53	82	10.25	3.8438
CIANCIO SJ, 2001, UROLOGY	10.1016/S0090-4295(00)01070-0	81	3.5217	1
FOWLER CJ, 1992, J NEUROL NEUROSURG PSYCHIATRY	10.1136/jnnp.55.11.986	73	2.2812	1.7176
MCCLURG D, 2008, NEUROUROL URODYN	10.1002/nau.20486	68	4.25	3.0357
GIANNANTONI A, 1999, ARCH PHYS MED REHABIL	10.1016/S0003-9993(99)90282-4	58	2.32	0.6
BRADLEY WE, 1978, NEUROLOGY	10.1212/wnl.28.9_part_2.52	51	1.1087	1
PETERSEN T, 1984, ACTA NEUROL SCAND	10.1111/j.1600-0404.1984.tb07823.x	49	1.225	1.9216
ÇETINEL B, 2013, NEUROUROL URODYN	10.1002/nau.22374	42	3.8182	2.1132
ENGELER DS, 2015, BMC UROL	10.1186/s12894-015-0102-x	35	3.8889	2.0792
DEL POPOLO G, 2008, NEUROL SCI	10.1007/s10072-008-1042-y	35	2.1875	1.5625
DE RIDDER D, 2013, CLIN NEUROL NEUROSURG	10.1016/j.clineuro.2013.06.018	32	2.9091	1.6101
LEMACK GE, 2005, UROLOGY	10.1016/j.urology.2004.11.038	32	1.6842	1.3913
SLIWA JA, 1996, ARCH PHYS MED REHABIL	10.1016/S0003-9993(96)90106-9	32	1.1429	1
CASTEL-LACANAL E, 2015, NEUROUROL URODYN	10.1002/nau.22495	31	3.4444	1.8416
TORNIC J, 2017, CURR NEUROL NEUROSCI REP	10.1007/s11910-018-0857-z	30	4.2857	1.8
HABEK M, 2010, CLIN NEUROL NEUROSURG	10.1016/j.clineuro.2010.04.010	29	2.0714	1
